# Bladder Pain Syndome/Interstitial Cystitis due to Pudendal Nerve Compression: Described in 1915—A Reminder for Treating Pelvic Pain a Century Later

**DOI:** 10.1055/s-0039-1700538

**Published:** 2020-03-06

**Authors:** Andreas Gohritz, Arnold Lee Dellon

**Affiliations:** 1 Department of Plastic, Reconstructive and Aesthetic Surgery, Hand Surgery, University Hospital, Basel, Switzerland; 2 Department of Plastic Surgery and Neurosurgery, Dellon Institutes for Peripheral Nerve Surgery, Johns Hopkins University, Baltimore, Maryland, United States

**Keywords:** bladder pain syndrome, interstitial cystitis, pudendal nerve, compression, Georg Zülzer

## Abstract

**Background** Interstitial cystitis (IC) or bladder pain syndrome (BPS) is highly painful and disabling and probably the most misdiagnosed urologic condition. Its classic symptoms of perineal pain, urinary urgency, and frequency despite sterile urine cultures were already described more than a century ago in a report on soldiers during World War (WW) I due to chronic pudendal nerve compression.

**Objectives** This article translates a report from 1915 on pudendal neuropathy and discusses its author Georg Zülzer (1870–1949).

**Methods** An English translation of the German original is provided with the biography and work of Zülzer, his clinical observations are discussed regarding modern diagnosis and therapy of pudendal nerve compression.

**Results** In his article entitled “Irritation of the Pudendal Nerve (Neuralgia). A Frequent Clinical Picture during War Feigning Bladder Catarrh,” Zülzer describes his observation of soldiers during WW I, presenting with a triad of perineal pain, urinary urgency, and frequency despite sterile urine cultures excluding urinary infections. He also documented a characteristic skin hypersensibility of the perineum in a rhomboid shape which corresponds to the innervation area of the pudendal nerve with its two branches deriving from the “pudendal plexus.” He regards this symptomology as rare during peace, but as disease of trench warfare which can be easily diagnosed regarding clear urine and a painful skin island overlying the area of the pudendal nerve as tested by simple needle examination. Zülzer, born in Germany, was forced to emigrate to the United States in 1934, was also an important pioneer of diabetes research using pancreas extracts from dogs as early as 1907.

**Conclusion** In this historical description, dating from about a century ago, Georg Zülzer probably gave the first exact clinical description of symptoms due to pudendal nerve compression. Pudendal nerve compression should always be taken into account when examining and treating patients with symptoms of IC/BPS.

## Introduction

Peripheral nerve compression, above all of the pudendal nerve, is still frequently overlooked in the differential diagnosis of pelvic pain even, in recent reviews. 1

## Objectives

The objective of this article is, on the occasion of the centenary of an article entitled “Irritation of the Pudendal Nerve (Neuralgia). A Frequent Clinical Picture during War Feigning Bladder Catarrh,” to make this very early report on pudendal nerve compression accessible for English-speaking physicians and therapists caring for individuals affected by pain related to the pudendal nerve and to discuss its author Georg Zülzer (1870–1949).

## Methods

An English translation of the original German report is provided together with a summary on the biography and work of its author. His clinical observations are discussed regarding modern diagnosis and therapy of pudendal nerve compression.

## Results

### Translation of the Original Report

In 1915, Zülzer, then head physician of a military hospital during World War (WW) I, published the following observations in a German medical journal 2 :

“A clinical picture, which I have experienced only twice in practice during peace, but which is occurring frequently (also confirmed to me from other side) during war and whose diagnosis is not always made correctly, is the irritation of the pudendal nerve. The patients report, in a consistent manner, an extraordinarily frequent urge to urinate, combined with a painful pressure in the bladder area and pain during urinating. The urine is without exception clear, sour, free from protein, without sediment. The singularly voided portions of urine are small. They vary between about 20 and 100 ccm. That only single drops are voided, as in acute bladder catarrh, does not occur. The mentioned clinical symptoms distinguished the addressed ailment from a bladder catarrh. If one tries to define the area of pain with the needle, as I had recommended in my paper on spinalgia for all diseases accompanied with pain, it was possible in all cases observed by me, about a number of 10 to 12, to document that a skin area which delimits the perineum in a rhomboid shape, shows an extraordinary hypersensibility. The front lace of the rhomboid is located 2 to 3 finger widths above the symphysis. The rear lace is located a little bit below the sacrum, approximately in the middle of the nates (clunium), about at the level of the after. The lateral laces are located lateral to the perineum, about the width of a hand, at the inner side of the thigh. This skin area corresponds to the skin area of the pudendal nerve with his two branches deriving from the pudendal plexus. The subjectively experienced, paroxysmally occurring (and always imitating urgency to urinate) complaints and the proof, that the skin hypersensibility is belonging to a defined nerve leave no doubt about the diagnosis, irritation of the pudendal nerve. If this irritation can be labelled as neuralgic, as I have done in the mentioned communication about the intercostal and other nerves appears of minor importance. The therapy of the ailment is, however, that of neuralgia, antineuralgica, as pyramidon, aspirin or alike, and local heat (hot air apparatus). Under this therapy, I could heal the ailment in a few cases in 6 to 10 days; not only the urge to urinate disappeared, but also the needle hypersensibility.

A short medical history shall be presented as a paradigma; Joseph J., 30 years old. Formerly always been healthy, he was suffering since 14 days from urge to urinate and pain during urinating, pressure in the bladder. He has to leave urine 40 to 50 times per 24 hours. During the last 24 hours, he has lost urine spontaneously. He has no explanation for the cause of the disease. Status: strong man, inner organs without peculiarities, urine clear, sour, free of protein and sugar, without sediment. The bladder does not seem to be filled by percussion. the needle examination reveals the above sketched rhomboid. Treatment: local hot air treatment, pyramidon thrice daily. After 5 days, significant improvement observed. During the last 24 hours only approximately 10 times of urinating. After 10 days, no hypersensibility anymore in the area of the pudendal nerve. Without complaints released to the troupe as healed.

The pudendal nerve irritation, which is very rare, as I have mentioned in my former observation, can be regarded as disease of the trench. Its diagnosis is possible in the field without problems, even under the simplest conditions, if you only take into account the macroscopically clear urine on the one side and on the other side if you document the bladder sensibility through the characteristically limited hypersensibility of the skin area of the pudendal nerve by simple needle examination. Recently, I also saw this neuralgia occurring in a case of typhus by the way.”

### Short Biography and Work of Georg Zülzer (1870–1949)

**Fig. 1:**
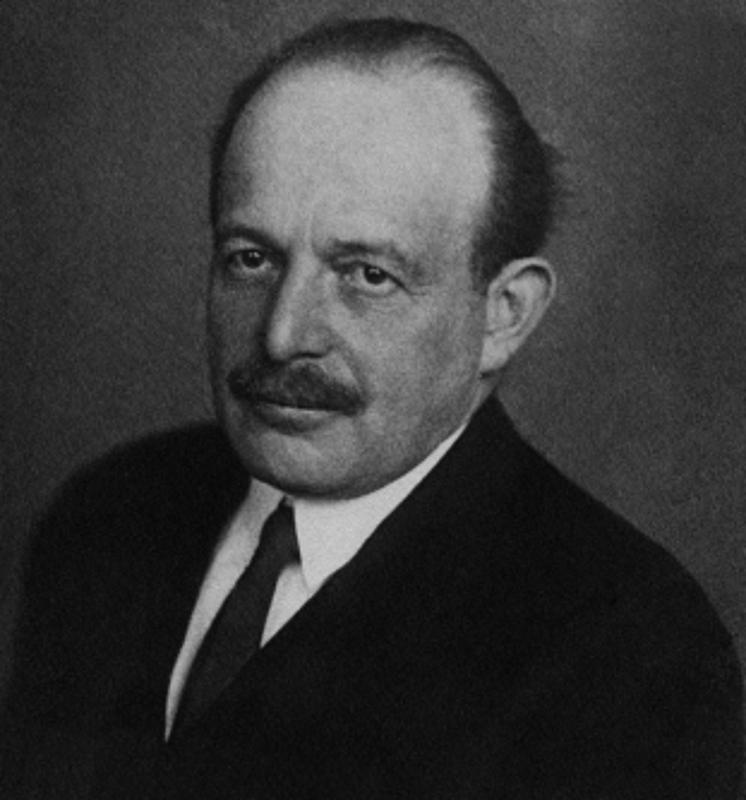
Georg Ludwig Zülzer (1870–1949).

Georg Ludwig Zülzer ( Fig. 1 ) was a German physician practicing in Berlin who emigrated to New York City in 1934, because he was discriminated as a Jew. He pioneered diabetes mellitus research treating diabetic dogs with extracts of calf pancreas and, in 1906, treating a patient dying from diabetic coma with an extract called “Acomatrol.” The patient first showed improvement, but later suffered from side effects, and died when the supply of Zülzer's pancreatic extract was exhausted. Zülzer continued to seek a suitable remedy for diabetes mellitus, but his laboratory was closed by the German military during WW I. A breakthrough occurred in the early 1920s when Canadian physicians Frederick Banting (1891–1941) and Charles Best (1899–1978) developed an extract that saved the life of a 14-year-old diabetic patient. They received the Nobel prize in 1923, although they acknowledged that their initial clinical trials were as “not so encouraging as those obtained by Zuelzer in 1908.” 3 4

## Discussion

As summarized in a recent review, interstitial cystitis or bladder pain syndrome (IC/BPS) is a chronic, severely painful, and disabling disease of unknown origin and probably the most misdiagnosed urologic condition affecting millions of patients. 1 5 Treatment is often frustrating and pain may be unresponsive even to removal of pelvic organs. 6 Classic symptoms include perineal pain, urinary urgency, and frequency despite sterile urine cultures, exactly the same as described by Zülzer in his soldier patients during WW I in whom he diagnosed pudendal nerve compression due to a constant cutaneous hypersensitivity in its anatomical area of innervation. Interestingly, research pioneered by Antolak and others newly supports a link between interstitial cystitis and pudendal nerve compression with overlapping symptoms including pelvic pain, typically in the perineal, rectal, and genital area, sexual and voiding dysfunction, difficulty with defecation and a feeling that a foreign object may be in the body. 7 Newly described as “cyclist's syndrome,” “pudendal canal syndrome,” or “Alcock's syndrome,” pudendal nerve compression can be triggered by chronic or acute pressure to the sitting area, repeated vaginal infections and chronic constipation, as well as secondary trauma due to childbirth, surgery, and biomechanical abnormalities (e.g., sacroiliac joint dysfunction or pelvic floor dysfunction). 8 9 As in IC, diagnosis is made by ruling out other causes, for example, from urology or gynecology and identification of sensory abnormality of the pudendal nerve, in a rhomboid, from above the symphysis pubis to the middle of sacrum and to the lateral borders of the perineum as already defined by Zülzer in 1915. Testing may be done by pinprick, neurophysiologic, or neurosensory testing with the Pressure-Specified Sensory Device as first applied by Hruby et al who diagnosed compression of the dorsal branch of the pudendal nerve in men in 2009. 10 X-ray-guided perineural injections may relieve or eliminate symptoms, 11 12 carefully selected patients can benefit from surgical decompression of the pudendal nerve, and its branches or neurectomy, if the nerve has been directly injured and a neuroma is present with high success rates. 10 13 14 15 16 17

Although Zülzer used the term “pudendal neuralgia,” in this interpretation of his writings, the preferred peripheral nerve terminology of either pudendal nerve “compression” or “neuroma” of a particular branch of the pudendal nerve is used to clarify pathophysiology and surgical treatment options. 18

For conjecture, one can ask why trench warfare might predispose to chronic pudendal nerve compression. A hypothesis is that the pudendal nerve crosses from posterior to anterior within the fascia of the obturator internus, making it susceptible to increased intrapelvic pressure. Increased intrapelvic pressure would result in decreased pudendal nerve blood flow, leading acutely to ischemic symptoms, and, in the long term, chronic scarring along the course of the pudendal nerve. Increased standing, straining to have a bowel movement, changes in pelvic pressure related to bladder pain, and perhaps sitting for prolonged periods with wet clothing about the ischial tuberosity may each contribute to some degree to increase the intrapelvic pressure upon the pudendal nerve. This hypothesis lends itself to clinical investigation by measuring and the correlating either intravaginal or intrarectal pressures as a proxy for intrapelvic pressure and correlating that with symptoms related to pudendal nerve compression. 16 17

As a take-home message taken from the inspiring historical description by Georg Zülzer dating from exactly 100 years ago, pudendal nerve compression should always be taken into account when examining and treating patients with symptoms of IC/BPS.
